# 4E-BP1 expression in embryonic postmitotic neurons mitigates mTORC1-induced cortical malformations and behavioral seizure severity but does not prevent epilepsy in mice

**DOI:** 10.3389/fnins.2023.1257056

**Published:** 2023-08-23

**Authors:** Lena H. Nguyen, Manas Sharma, Angelique Bordey

**Affiliations:** ^1^Department of Neuroscience, School of Behavioral and Brain Sciences, University of Texas at Dallas, Richardson, TX, United States; ^2^Departments of Neurosurgery and Cellular & Molecular Physiology, Wu Tsai Institute, Yale University School of Medicine, New Haven, CT, United States

**Keywords:** mTOR, 4E-BP, seizures, epilepsy, corticogenesis, cortical development, malformation of cortical development

## Abstract

Hyperactivation of the mechanistic target of rapamycin complex 1 (mTORC1) pathway during neurodevelopment leads to focal cortical malformations associated with intractable seizures. Recent evidence suggests that dysregulated cap-dependent translation downstream of mTORC1 contributes to cytoarchitectural abnormalities and seizure activity. Here, we examined whether reducing cap-dependent translation by expressing a constitutively active form of the translational repressor, 4E-BP1, downstream of mTORC1 would prevent the development of cortical malformations and seizures. 4E-BP1^CA^ was expressed embryonically either in radial glia (neural progenitor cells) that generate cortical layer 2/3 pyramidal neurons or in migrating neurons destined to layer 2/3 using a conditional expression system. In both conditions, 4E-BP1^CA^ expression reduced mTORC1-induced neuronal hypertrophy and alleviated cortical mislamination, but a subset of ectopic neurons persisted in the deep layers and the white matter. Despite the above improvements, 4E-BP1^CA^ expression in radial glia had no effects on seizure frequency and further exacerbated behavioral seizure severity associated with mTORC1 hyperactivation. In contrast, conditional 4E-BP1^CA^ expression in migratory neurons mitigated the severity of behavioral seizures but the seizure frequency remained unchanged. These findings advise against targeting 4E-BPs by 4E-BP1^CA^ expression during embryonic development for seizure prevention and suggest the presence of a development-dependent role for 4E-BPs in mTORC1-induced epilepsy.

## 1. Introduction

Hyperactivation of the mechanistic target of rapamycin complex 1 (mTORC1) signaling pathway during embryonic development leads to focal malformation of cortical development (FMCD) and epilepsy (Crino, 2015). The mTORC1 pathway is a key regulator of cell growth and controls a wide range of cellular processes, including protein synthesis, autophagy, and metabolism (Laplante and Sabatini, 2012). The best-characterized function of mTORC1 is the control of cap-dependent translation via eukaryotic translation initiation factor 4E (eIF4E)-binding proteins (4E-BPs) (Richter and Sonenberg, 2005; Ma and Blenis, 2009). mTORC1 promotes cap-dependent translation by directly phosphorylating and inactivating the translational repressors, 4E-BP1 and 4E-BP2. Phosphorylated 4E-BPs dissociate from eIF4E, allowing eIF4E to form the eIF4F complex and initiate translation. Accumulating evidence has supported a critical role for 4E-BP-eIF4E-mediated translation in the mechanism of mTORC1-induced FMCD pathology and epilepsy. Several studies have shown increased levels of phosphorylated 4E-BPs in human and mouse FMCD brain tissue (Baybis et al., 2004; Orlova and Crino, 2010; Hanai et al., 2017; Kim et al., 2019; Nguyen et al., 2022). Furthermore, genetic and pharmacological inhibition of eIF4E in mice expressing mTORC1-activating gene variants rescued FMCD pathologies and seizures (Kim et al., 2019). Consistent with a role for 4E-BP-eIF4E signaling in the disease pathogenesis, embryonic expression of a constitutive active 4E-BP1 mutant (4E-BP1^CA^) that resists mTORC1 phosphorylation (Choi et al., 2003; Schalm et al., 2003) rescued several FMCD pathologies, including cortical mislamination and neuronal overgrowth, in mice expressing constitutive active Rheb (Rheb^CA^; the direct activator of mTORC1) (Gong et al., 2015; Lin et al., 2016). Additionally, expression of 4E-BP1^CA^ in adolescent Rheb^CA^ mice, after the onset of seizures, alleviated the seizure frequency and associated FMCD pathologies (Nguyen et al., 2022). Considering that FCMDs are formed during cortical neurogenesis and can be detected during fetal development in humans (Glenn et al., 2012; Lerman-Sagie et al., 2021), earlier therapeutic interventions could potentially limit FCMD formation and seizure development. However, the effects of embryonic 4E-BP1^CA^ expression on seizure development remain unknown.

In this study, we examined whether embryonic expression of 4E-BP1^CA^ in cortical pyramidal neurons prevents mTORC1-induced seizures using the Rheb^CA^ mouse model. We co-expressed Rheb^CA^ with 4E-BP1^CA^, which was previously shown to reduce cap-dependent translation (Lin et al., 2016; Nguyen et al., 2022), in developing cortices via *in utero* electroporation (IUE). We used two experimental paradigms. In the first condition, 4E-BP1^CA^ was expressed in radial glia (neural progenitor cells) that generate cortical layer (L) 2/3 pyramidal neurons. In the second condition, we bypassed radial glia and selectively expressed 4E-BP1^CA^ in postmitotic migrating neurons destined to L2/3 through a conditional expression system. In both conditions, 4E-BP1^CA^ expression reduced mTORC1-induced neuronal hypertrophy and alleviated cortical mislamination by increasing the proportion of correctly positioned neurons in the cortex. Despite these histological improvements, 4E-BP1^CA^ expression in radial glia had no effects on seizure frequency and further exacerbated behavioral seizure severity in Rheb^CA^ mice. In contrast, selective 4E-BP1^CA^ expression in postmitotic migratory neurons mitigated the severity of behavioral seizures but the seizure frequency remained unchanged. These findings caution against targeting 4E-BPs by 4E-BP1^CA^ expression during embryonic development for seizure prevention and suggest the presence of a developmentally dependent role of 4E-BPs in mTORC1-induced epilepsy.

## 2. Materials and methods

### 2.1. Animals

All animal procedures were performed in accordance with Yale University Institutional Animal Care and Use Committee’s regulations. All experiments were performed on male and female CD-1 mice (Charles River Laboratories).

### 2.2. *In utero* electroporation (IUE)

IUE was performed in timed-pregnant embryonic day (E) 15 mice as previously described (Nguyen et al., 2022). Briefly, a solution of the appropriate DNA plasmid mixture was injected into the right lateral ventricle of each embryo using a glass pipette. All solutions contained 0.03% Fast Green dye to visualize the injection. For each litter, half of the embryos were injected with the experimental plasmids and the other half received the respective control plasmids. Following each injection, a 5 mm tweezer electrode was positioned on the embryo’s head and six 42 V, 50 ms pulses at 950 ms intervals were applied using a pulse generator (ECM830, BTX). The electrodes were positioned to target expression in the medial prefrontal cortex (mPFC). Pups were screened at postnatal day (P) 0 to verify successful electroporation to the targeted region by detecting fluorescent reporter expression (tdTomato or GFP) on a fluorescence-enabled stereomicroscope.

In the first experimental paradigm (Rheb^CA^ + 4E-BP1^CA^), mice were electroporated with a plasmid mixture consisting of pCAG-Rheb^CA^ + pCAG-4E-BP1^CA^ (or pCAG-GFP as control) + pCAG-tdTomato. In the second condition (Rheb^CA^ + conditional [c]4E-BP1^CA^), mice were electroporated with a plasmid mixture consisting of pCAG-Rheb^CA^+ pCALNL-4E-BP1^CA^ (or pCALNL-DsRed as control) + pCIG2-DCX-Cre-IRES-GFP + pCALNL-GFP. The plasmid concentrations and sources are listed in Table 1.

**Table 1:** DNA plasmid information.

Plasmid	Concentration (μg/μl)	Source
pCAG- Rheb^CA^ (Rheb^S16H^)	2.5	Gift from Drs. Tomohiko Maehama and Kentaro Hanada (National Institute of Infectious Diseases, Tokyo, Japan) (Maehama et al., 2008)
pCAG-4E-BP1^CA^ (4E-BP1^F113A^)	3.0	Addgene #81122; the F113A mutation resists phosphorylation by mTORC1 and constitutively binds to eIF4E (Choi et al., 2003; Schalm et al., 2003)
pCAG-GFP	3.0	Addgene #11150
pCAG-tdTomato	0.8	Addgene #83029
pCALNL-4E-BP1^CA^ (4E-BP1^F113A^) (c4E-BP1^CA^)	3.0	Generated as previously described (Nguyen et al., 2022)
pCALNL-DsRed (cDsRed)	3.0	Addgene #13769
pCALNL-GFP (cGFP)	0.9	Addgene #13770
pCIG2-DCX-Cre-GFP	0.9–1.0	Gift from Dr. Ulrich Mueller (Scripps Institute, La Jolla, CA, United States) (Franco et al., 2011)

### 2.3. Video-EEG recording and analysis

Mice were implanted at 9–10 (Rheb^CA^ + 4E-BP1^CA^) or 6–8 (Rheb^CA^ + c4E-BP1^CA^) weeks of age with prefabricated EEG headmounts (Pinnacle Technology, Inc.) for subdural cortical EEG recording as previously described (Nguyen et al., 2019, 2022). Mice were video-EEG recorded starting at 10–12 (Rheb^CA^ + 4E-BP1^CA^) or 7–8 (Rheb^CA^ + c4E-BP1^CA^) weeks of age, during which they were housed in individual recording chambers in a light-, temperature-, and humidity-controlled room with *ad libitum* access to food and water. Synchronous video-EEG recording was acquired using a three-channel EEG tethered system and Sirenia Acquisition software (Pinnacle Technology, Inc.). Mice were recorded 24 h/day for at least 7 consecutive days. The average recording hours per animal was 173.1 ± 18.9 SD (Rheb^CA^ + 4E-BP1^CA^) and 170.7 ± 13.4 SD (Rheb^CA^ + c4E-BP1^CA^) hours.

Seizure frequency and severity were analyzed using Sirenia Seizure Basic software (Pinnacle Technology, Inc.). All analyses were performed blinded to experimental groups. The entire EEG traces were manually reviewed for the occurrence of electrographic seizures, defined as a sudden onset of high amplitude activity with a characteristic pattern of progressive frequency and amplitude increases over the course of the event lasting ≥10 s (Nguyen et al., 2015, 2019). The mean number of seizures per day (seizures/day) was obtained by the equation: (total number of seizures/total recording hours) × 24 for each animal. For each identified seizure, synchronous video recordings were visually inspected, and the severity of the behavioral seizure correlates was scored using a modified Racine scale (Table 2).

**Table 2:** Modified Racine scale.

Grade	Behavior
0	No change in behavior
1	Sudden behavioral arrest, facial clonus
2	Myoclonic jerks, head nodding, tonic elongation or twisting of the trunk, backward scooping or circling
3	Single forelimb clonus, straub tail
4	Both forelimb clonus, tonic–clonic seizure (without rearing)
5	Tonic–clonic seizure (with rearing or falling)

### 2.4. Histology

Brain tissue was collected for histological analysis at the end of EEG recording. Mice were deeply anesthetized with sodium pentobarbital (85 mg/kg i.p. injection) and perfused with ice-cold phosphate buffered saline (PBS, in mM: 137 NaCl, 2.7 KCl, 4.3 Na_2_HPO_4_, 1.47 KH_2_PO_4_, pH 7.4) followed by 4% paraformaldehyde (PFA). Whole brains were dissected and post-fixed in 4% PFA for 2 h and then cryoprotected in 30% sucrose for 24–48 h at 4°C. Brains were cut into 50 μm-thick coronal sections using a freezing microtome and stored in PBS + 0.01% sodium azide at 4°C until use. Sections were mounted onto glass slides and coverslipped with antifade mounting media before imaging.

Images were acquired using an Olympus Fluoview FV1000 confocal microscope and analyzed with ImageJ software (NIH). Representative images for figures were prepared using Adobe Photoshop CC. All images meant for direct comparison were taken with the same microscope settings and uniformly processed. All image analyses were performed blinded to the experimental groups. Cell size was quantified from 20X magnification images by tracing around the soma of tdTomato+ or GFP+ cells, as appropriate for each experimental condition, and measuring the area. 20–50 randomly selected cells from 2–3 brain sections were measured and averaged for each animal. Cell placement was quantified from 10X magnification images within a region of interest (ROI) spanning from the white matter border to the pial surface. The ROI height was set to 150 μm and the length was defined by the distance from the peak of the cingulum bundle to pia. The ROI was divided into 10 equal vertical bins, and the number of tdTomato+ or GFP+ cells in each bin was counted. Data are shown as % of total tdTomato+ or GFP+ cells within the ROI. 2 brain sections were analyzed and averaged for each animal.

### 2.5. Statistical analysis

All statistical analyses were performed using GraphPad Prism 9 software, except for Pearson’s Chi-Squared test and the accompanying *z*-tests, which were performed using IBM SPSS 26.0 software. The specific tests and sample sizes (*n*, number of animals) are described in the results and figure legends. The significance level was set at *p* < 0.05. Data are presented as mean ± standard error of the mean (SEM).

## 3. Results

### 3.1. 4E-BP1^CA^ expression in L2/3 pyramidal neurons by targeting radial glia has no effects on seizure frequency and exacerbates behavioral seizure severity in Rheb^CA^ mice

**Figure 1:**
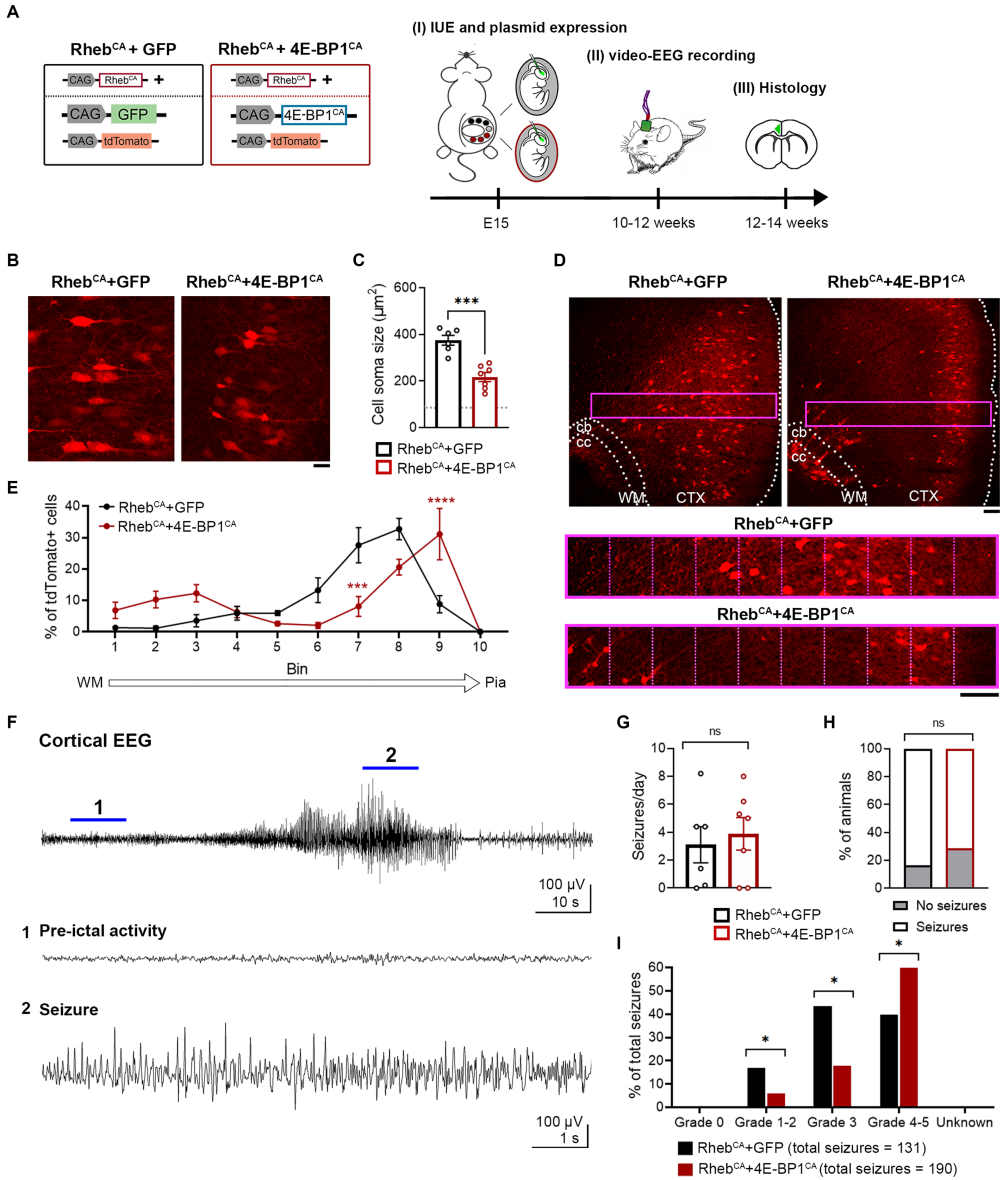
4E-BP1^CA^ expression (by embryonic targeting of radial glia) decreases neuron hypertrophy, improves cortical lamination, and exacerbates behavioral seizure severity in Rheb^CA^ mice. **(A)** Schematic diagram of plasmids used for IUE and experimental timeline. **(B)** Representative images showing tdTomato+ cells in electroporated cortices in 12-week-old Rheb^CA^ + GFP and Rheb^CA^ + 4E-BP1^CA^ mice. Scale bar = 25 μm. **(C)** Quantification of tdTomato+ cell soma size. Each data point represents averaged values from 50 cells/animal. Data were analyzed using unpaired *t*-test. Gray dotted line shows previously reported levels for adult control mice electroporated with GPF for visual comparison (Nguyen et al., 2019). **(D)** Representative images showing tdTomato+ cell placement in electroporated cortices in 12-week-old Rheb^CA^ + GFP and Rheb^CA^ + 4E-BP1^CA^ mice. A defined region of the cortex (purple rectangle, enlarged on the bottom) spanning from the WM border to pia was divided into 10 equal bins, and the % of cells in each bin was quantified to assess cell distribution. Scale bars = 100 μm. **(E)** Quantification of tdTomato+ cell placement in the cortex. Each data point represents averaged values from 2 brain sections/animal. Data were analyzed using two-way repeated measures ANOVA with Šídák’s post-hoc test. **(F)** Representative EEG trace showing a typical electrographic seizure in an 11-week-old Rheb^CA^ + GFP mouse. Expanded traces from the region indicated by the blue lines are shown at the bottom. **(G)** Quantification of seizure frequency. Each data point represents mean seizures/day from 7 days per animal. Data were analyzed using Mann–Whitney U test. **(H)** Quantification of seizure incidence. Data were analyzed using Fisher’s Exact test. **(I)** Quantification of behavioral seizure severity based on a modified Racine scale. Data were analyzed using Pearson’s Chi-Squared test with accompanying *z*-tests to compare proportions. **(C,E,G–I)** *n* = 6 Rheb^CA^ + GFP, 7 Rheb^CA^ + 4E-BP1^CA^ mice. *p < 0.05, ***p < 0.001, ****p < 0.0001. Error bars are ± SEM. IUE, in utero electroporation; CTX, cortex; WM, white matter; cb, cingulum bundle; cc, corpus callosum.

Increasing mTORC1 activity via Rheb^CA^ expression during corticogenesis in mice recapitulates human FMCD pathology, including neuronal hypertrophy and mispositioning in the cortex, and seizures (Hsieh et al., 2016; Lin et al., 2016; Nguyen et al., 2019). To examine whether embryonic 4E-BP1^CA^ expression would prevent Rheb^CA^-induced FMCD pathology and seizure onset, we co-electroporated Rheb^CA^ and 4E-BP1^CA^ in E15 cortices, targeting radial glia that gives rise to L2/3 pyramidal neurons (Figure 1A). Control mice were co-electroporated with Rheb^CA^ and GFP. A tdTomato reporter was included in both groups to label targeted cells.

4E-BP1^CA^ expression significantly decreased the soma size of Rheb^CA^ neurons and increased the proportion of correctly positioned neurons in L2/3 as examined in brain sections from adult mice (Figures 1B–E). However, we observed a subset of heterotopic Rheb^CA^ + 4E-BP1^CA^-expressing neurons that remained in the deep cortical layers, near the cingulum bundle, and in the white matter (Figure 1D). Despite the histological improvements, the frequency and incidence of seizures, as assessed by EEG recording, were unchanged between Rheb^CA^ + 4E-BP1^CA^ and Rheb^CA^ + GFP mice (Figures 1F–H). To determine whether the behavioral manifestations of seizures were improved, we scored the associated motor symptoms for all identified electrographic seizures using a modified Racine scale (Table 2). Surprisingly, we found a significant increase in the proportion of grade 4–5 seizures and a decrease in grades 1–2 and 3 seizures in Rheb^CA^ + 4E-BP1^CA^ compared to Rheb^CA^ + GFP mice (Figure 1I). These findings suggest that embryonic expression of 4E-BP1^CA^ in E15 radial glia generating L2/3 pyramidal neurons worsens Rheb^CA^-induced seizure severity.

### 3.2. Conditional 4E-BP1^CA^ expression in L2/3 pyramidal neurons by selective embryonic targeting of postmitotic migratory neurons mitigates behavioral seizure severity but not frequency in Rheb^CA^ mice

**Figure 2:**
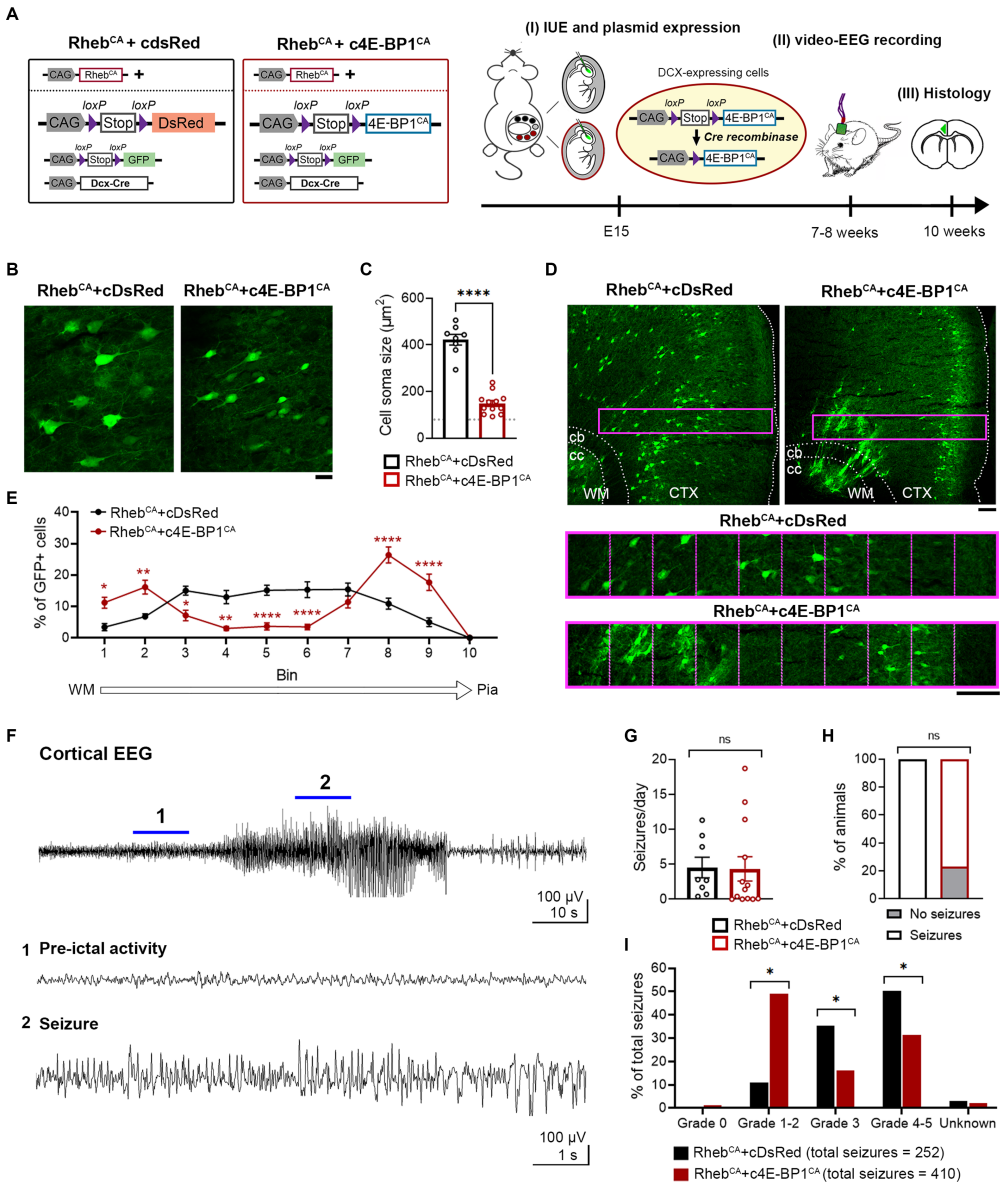
c4E-BP1^CA^ expression (by selective embryonic targeting of postmitotic migratory neurons) decreases neuron hypertrophy, improves cortical lamination, and mitigates behavioral seizure severity in Rheb^CA^ mice. **(A)** Schematic diagram of plasmids used for IUE and experimental timeline. **(B)** Representative images showing GFP+ cells in electroporated cortices in 10-week-old Rheb^CA^ + cDsRed and Rheb^CA^ + c4E-BP1^CA^ mice. Scale bar = 25 μm. **(C)** Quantification of GFP+ cell soma size. Each data point represents averaged values from 20 cells/animal. Data were analyzed using unpaired *t*-test. Gray dotted line shows previously reported levels for adult control mice electroporated with GPF for visual comaprison (Nguyen et al., 2019). **(D)** Representative images showing GFP+ cell placement in electroporated cortices in 10-week-old Rheb^CA^ + cDsRed and Rheb^CA^ + c4E-BP1^CA^ mice. A defined region of the cortex (purple rectangle, enlarged on the bottom) spanning from the WM border to pia was divided into 10 equal bins, and the % of cells in each bin was quantified to assess cell distribution. Scale bars = 100 μm. **(E)** Quantification of GFP+ cell placement in the cortex. Each data point represents averaged values from 2 brain sections/animal. Data were analyzed using two-way repeated measures ANOVA with Šídák’s post-hoc test. **(F)** Representative EEG trace showing a typical electrographic seizure in a 9-week-old Rheb^CA^ + cDsRed mouse. Expanded traces from the region indicated by the blue lines are shown at the bottom. **(G)** Quantification of seizure frequency. Each data point represents mean seizures/day from 7 days per animal. Data were analyzed using Mann–Whitney U test. **(H)** Quantification of seizure incidence. Data were analyzed using Fisher’s Exact test. **(I)** Quantification of behavioral seizure severity based on a modified Racine scale. Data were analyzed using Pearson’s Chi-Squared test with accompanying *z*-tests to compare proportions. **(C,E,G–I)** *n* = 8 Rheb^CA^ + cDsRed, 12–13 Rheb^CA^ + c4E-BP1^CA^ mice, *p < 0.05, **p < 0.01, ****p < 0.0001. Error bars are ± SEM. IUE, in utero electroporation; CTX, cortex; WM, white matter; cb, cingulum bundle; cc, corpus callosum.

Considering that the heterotopic neurons were not prevented by 4E-BP1^CA^ expression, we hypothesized that expression of 4E-BP1^CA^ in radial glia may indirectly affect the migration of a subset of pyramidal neurons. In addition, we speculated that mTORC1 activation due to Rheb^CA^ may lag behind 4E-BP1^CA^’s effect on cap-dependent translation, resulting in an inappropriate reduction of translation prior to mTORC1 hyperactivation. To address these issues, we bypassed radial glia and selectively targeted 4E-BP1^CA^ expression in postmitotic migratory neurons using a conditional Cre-lox system. We co-electroporated Rheb^CA^ with Cre-inducible, conditional 4E-BP1^CA^ (c4E-BP1^CA^) and Cre recombinase under the control of a doublecortin (DCX) promoter (DCX-Cre) (Figure 2A). In this experimental condition, c4E-BP1^CA^ expression is restricted to DCX+ positive migratory neurons whereas Rheb^CA^ is expressed in radial glia and subsequently neurons in the same manner as above. Rheb^CA^ is thus functionally expressed in newborn neurons before 4E-BP1^CA^ functional expression. Control mice were co-electroporated with Rheb^CA^, conditional DsRed (cDsRed), and DCX-Cre. A conditional GFP (cGFP) reporter was included in both groups to label targeted cells.

c4E-BP1^CA^ expression significantly decreased the soma size of Rheb^CA^ neurons and increased the proportion of correctly positioned neurons in L2/3 (Figures 2B–E). Similar to the 4E-BP1^CA^ condition described above (Figure 1D), we observed a subset of heterotopic neurons in the deep cortical layers and white matter; however, this was even more prominent than in the above condition and significantly different from the control group (Figures 2D,E). c4E-BP1^CA^ expression did not reduce the seizure frequency or incidence in Rheb^CA^ mice (Figures 2F–H). However, the behavioral symptoms associated with seizures were less severe in the Rheb^CA^ + c4E-BP1^CA^ compared to Rheb^CA^ + cDsRed mice, with a significantly higher proportion of seizures scored at Racine grade 1–2 and a lower proportion scored at grades 3 and 4–5 (Figure 2I). These findings suggest that selective embryonic expression of 4E-BP1^CA^ in postmitotic migrating neurons destined to L2/3 mitigates the severity of Rheb^CA^-induced behavioral seizures despite the persistent presence of neuronal heterotopia.

## 4. Discussion

In this study, we examined whether embryonic expression of 4E-BP1^CA^ prevents FMCD and seizure development in Rheb^CA^ mice with hyperactive mTORC1 signaling. We found that 4E-BP1^CA^ expression during corticogenesis improved FMCD pathology but did not prevent seizure onset nor reduced seizure frequency in mice. These findings underscore the risks of reducing cap-dependent translation through 4E-BP1^CA^ expression during fetal development, including in conditions where mTORC1 is hyperactivated.

Embryonic expression of 4E-BP1^CA^ by targeting E15 radial glia or by selectively targeting postmitotic migrating neurons both led to decreased neuronal hypertrophy, consistent with previous studies (Lin et al., 2016). However, these manipulations did not fully restore the neuron soma size to the published levels for normal controls (Nguyen et al., 2019), suggesting that other molecular players may contribute to this phenotype. Embryonic expression of 4E-BP1^CA^ also improved neuronal positioning. However, in both experimental paradigms, we observed a cluster of heterotopic neurons in the deep layer near the cingulum bundle and in the white matter, indicating that 4E-BP1^CA^ failed to rescue these heterotopias regardless of the timing of 4E-BP1^CA^ expression. Interestingly, these neuronal heterotopias were worse when 4E-BP1^CA^ was selectively expressed in the migrating neurons compared to that in radial glia. These findings suggest that these heterotopias likely do not result from defects in radial glia expressing 4E-BP1^CA^. Why some neurons remained stuck in the deep layer and white matter when most successfully reached their proper location following 4E-BP1^CA^ expression is unknown. Further investigation into the molecular and laminar identities of these neurons could provide insights into how they arise and why they persist after 4E-BP1^CA^ expression.

Despite the improvements in FMCD histology, 4E-BP1^CA^ expression targeting radial glia had no effect on seizure frequency and unexpectedly exacerbated the behavioral seizure intensity in Rheb^CA^ mice. In contrast, selective 4E-BP1^CA^ expression targeting migrating neurons mitigated the severity of behavioral seizures although the seizure frequency remained unchanged. The failure of embryonic 4E-BP1^CA^ expression to prevent or reduce seizures was surprising given that embryonic 4E-BP1^CA^ expression has been shown to improve many mTORC1-induced cellular phenotypes, including somatic hypertrophy and positioning, as reported here and elsewhere (Lin et al., 2016), as well as dendritic and axonal overgrowth (Gong et al., 2015; Lin et al., 2016), and postnatal expression of 4E-BP1^CA^ significantly reduced seizure frequency (Nguyen et al., 2022). Given that heterotopic neurons in the deep layer and white matter remained following 4E-BP1^CA^ expression, it is possible that these contribute to seizure generation. However, we found no correlations between these neuronal heterotopias and seizure severity across individual animals, and the heterotopias were notably worse in the c4E-BP1^CA^ condition that was associated with reduced seizure severity. Furthermore, given that such heterotopias and neuronal misplacement were present following adolescent 4E-BP1^CA^ expression that decreased seizure frequency (Nguyen et al., 2022), the presence of these heterotopias likely does not considerably account for the persistent seizures in Rheb^CA^ mice.

Another possible explanation for the persistent seizures following 4E-BP1^CA^ expression is that 4E-BP1 may have specific functions during embryonic cortical development (e.g., regulate the translation of specific subsets of developmentally relevant mRNAs), and altering these functions via 4E-BP1^CA^ expression could interfere with these processes, leading to unrepressed seizures, and in some cases, exacerbation of behavioral seizure severity. In line with this idea, genetic inhibition of mTORC1, and subsequently, reduction of 4E-BP phosphorylation, via embryonic deletion of *Raptor* in *Pten* knockout mice did not suppress seizures (Chen et al., 2019) despite rescuing brain size, neuronal overgrowth, and synapse function (Chen et al., 2019; Tariq et al., 2022). One study also reported an unexpected increase in the mortality of these mice (Cullen et al., 2023). Interestingly, embryonic knockdown of eIF4E successfully reduced the seizure frequency in mice expressing mTORC1-activating mutants (Kim et al., 2019). eIF4E is a binding partner of 4E-BP1 and required for cap-dependent translation (Richter and Sonenberg, 2005). Both 4E-BP1^CA^ expression and eIF4E knockdown reduce translation, and the reasons behind the diverging impacts on seizures are unclear. Overall, these results suggest a complicated, developmentally regulated role for 4E-BPs in mTOR-induced epilepsy.

Taken together, our study caution against 4E-BP1^CA^ expression in embryonic life for seizure prevention and suggest a complex developmental stage-dependent role for 4E-BPs in mTORC1-induced epilepsy. Although our study shows that embryonic 4E-BP1^CA^ expression does not prevent seizures, alternative approaches to repress translation, such as directly inhibiting the phosphorylation of endogenous 4E-BPs, may yield different results and should be further explored. Future studies aimed to dissect the functions of 4E-BPs in cortical development could shed additional light on these issues and provide insights into new treatment avenues for mTORC1-induced epilepsy.

## Data availability statement

The original contributions presented in the study are included in the article/supplementary material, further inquiries can be directed to the corresponding authors.

## Ethics statement

The animal study was approved by Yale University Institutional Animal Care and Use Committee (IACUC). The study was conducted in accordance with the local legislation and institutional requirements.

## Author contributions

LN: Conceptualization, Investigation, Formal Analysis, Visualization, Writing – original draft, Writing – review & editing, Funding acquisition. MS: Formal analysis. AB: Conceptualization, Writing – review & editing, Funding acquisition.

## Funding

The author(s) declare financial support was received for the research, authorship, and/or publication of this article. This work was supported by National Institutes of Health (NIH) F32 HD095567 (LN), R01 NS111980 (AB).

## Conflict of interest

LN and AB are co-inventors on a patent application, PCT/US2020/054007 entitled “Targeting Cap-Dependent Translation to Reduce Seizures in mTOR disorders”. 2021-04-08: Publication of WO2021067752A1.

The remaining author declares that the research was conducted in the absence of any commercial or financial relationships that could be construed as a potential conflict of interest.

## Publisher’s note

All claims expressed in this article are solely those of the authors and do not necessarily represent those of their affiliated organizations, or those of the publisher, the editors and the reviewers. Any product that may be evaluated in this article, or claim that may be made by its manufacturer, is not guaranteed or endorsed by the publisher.
